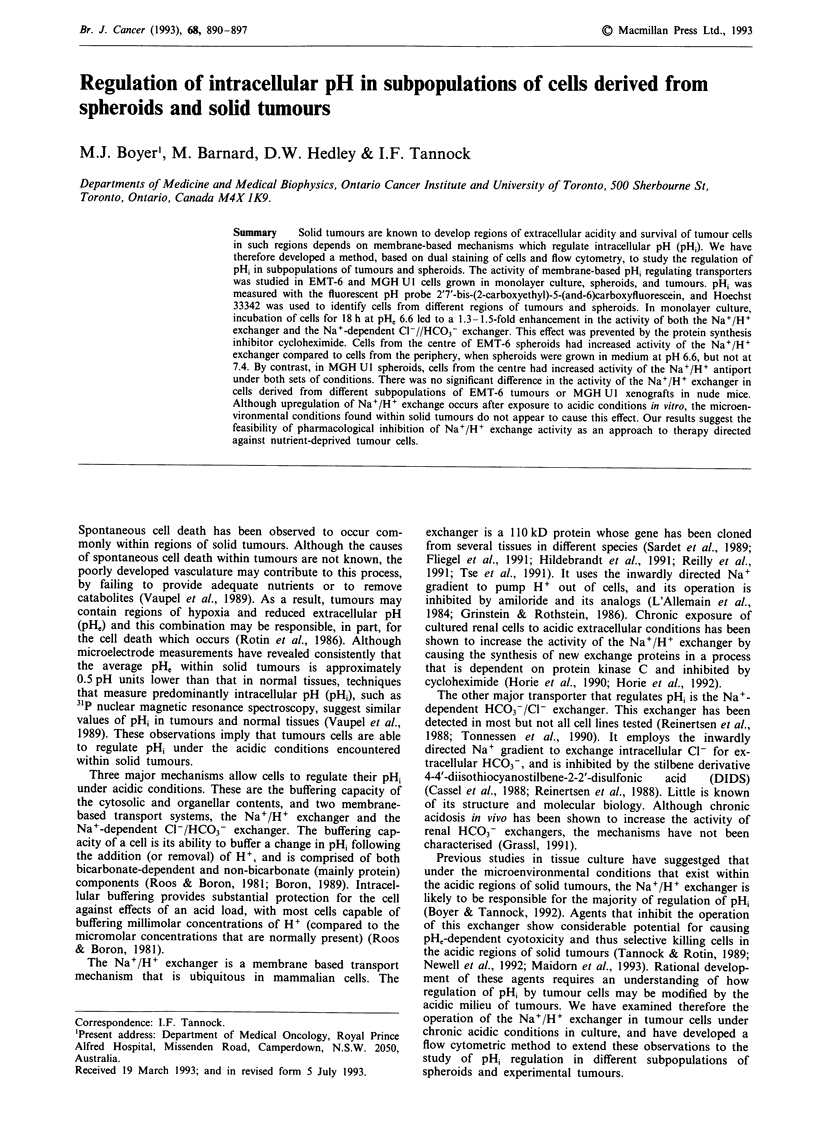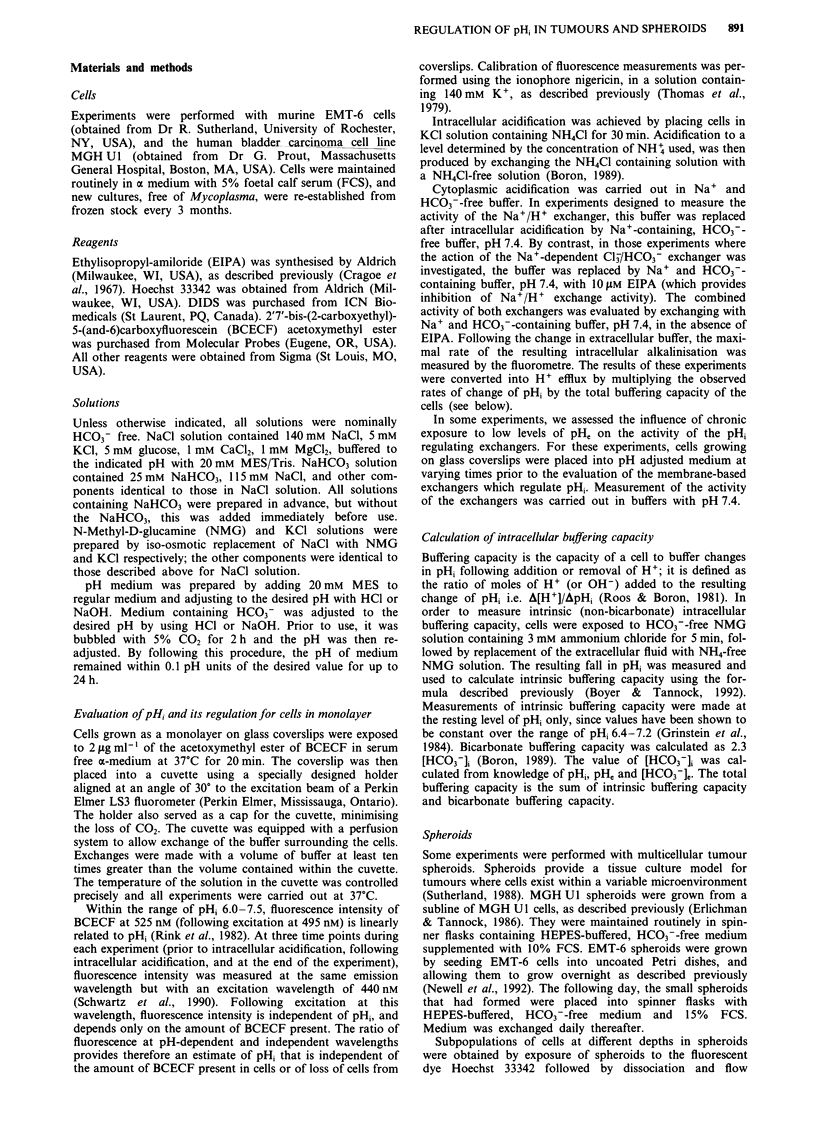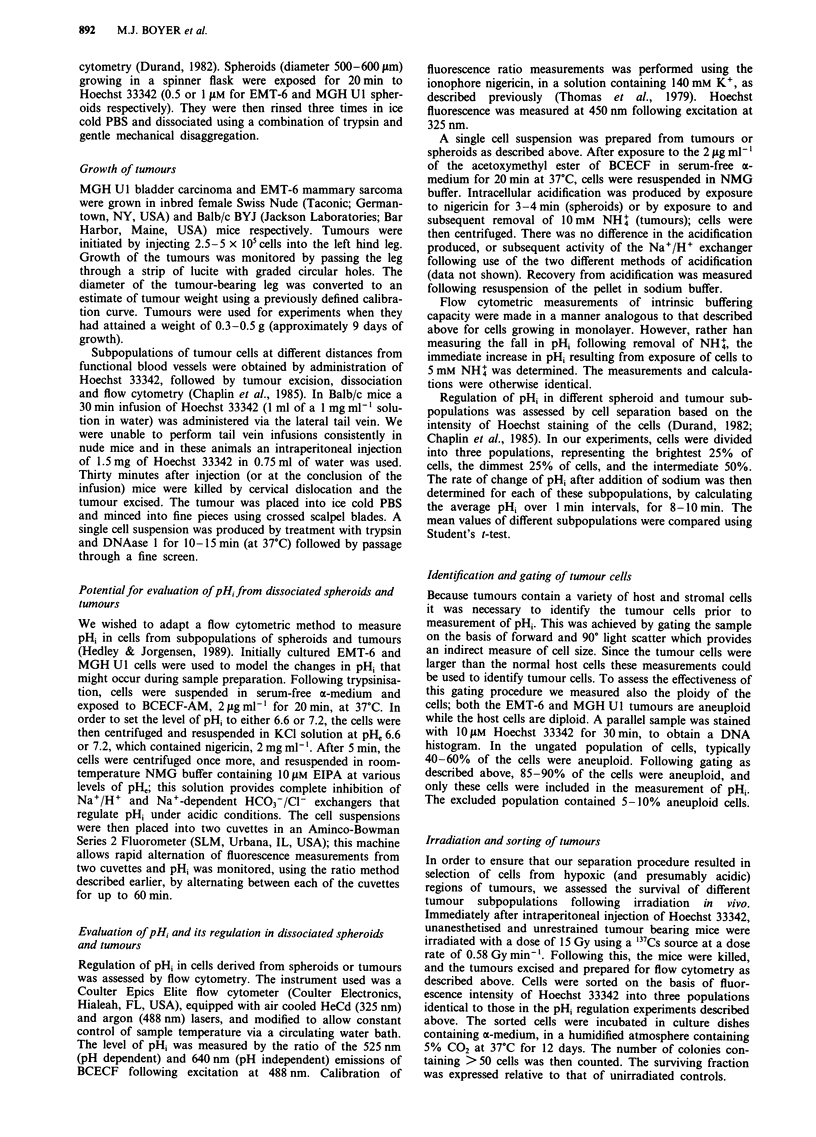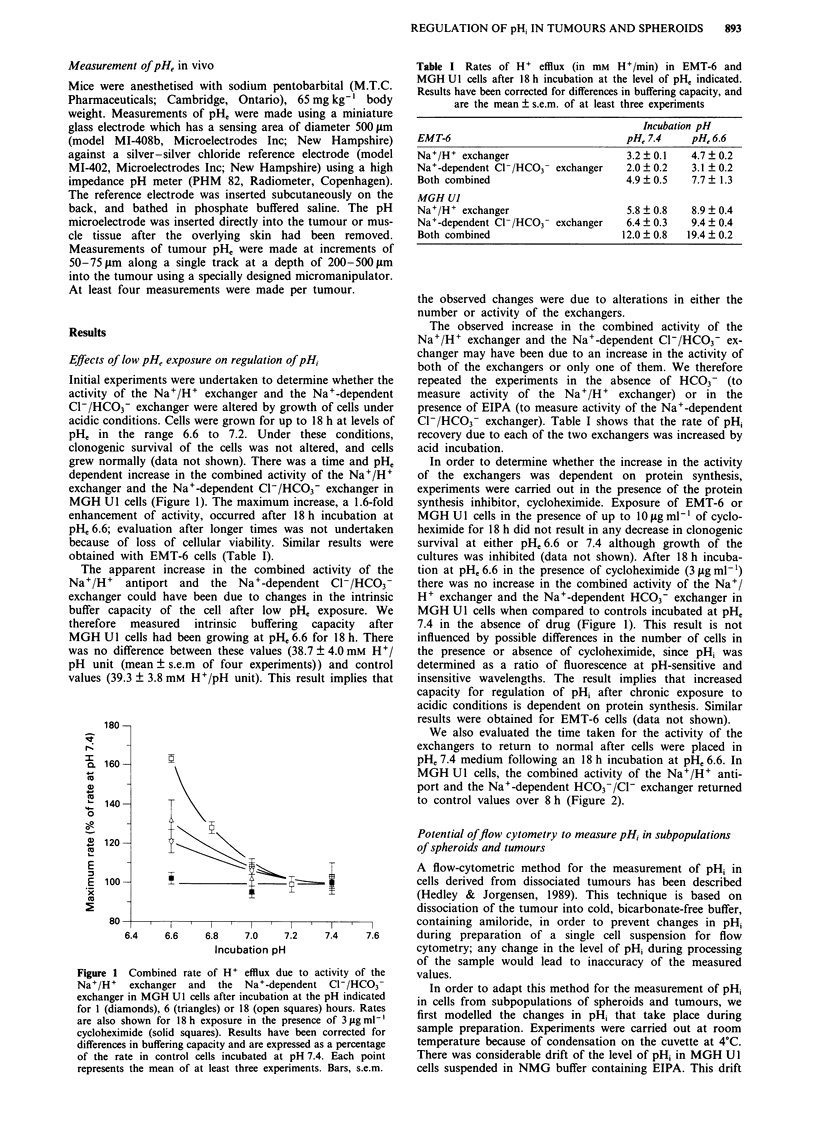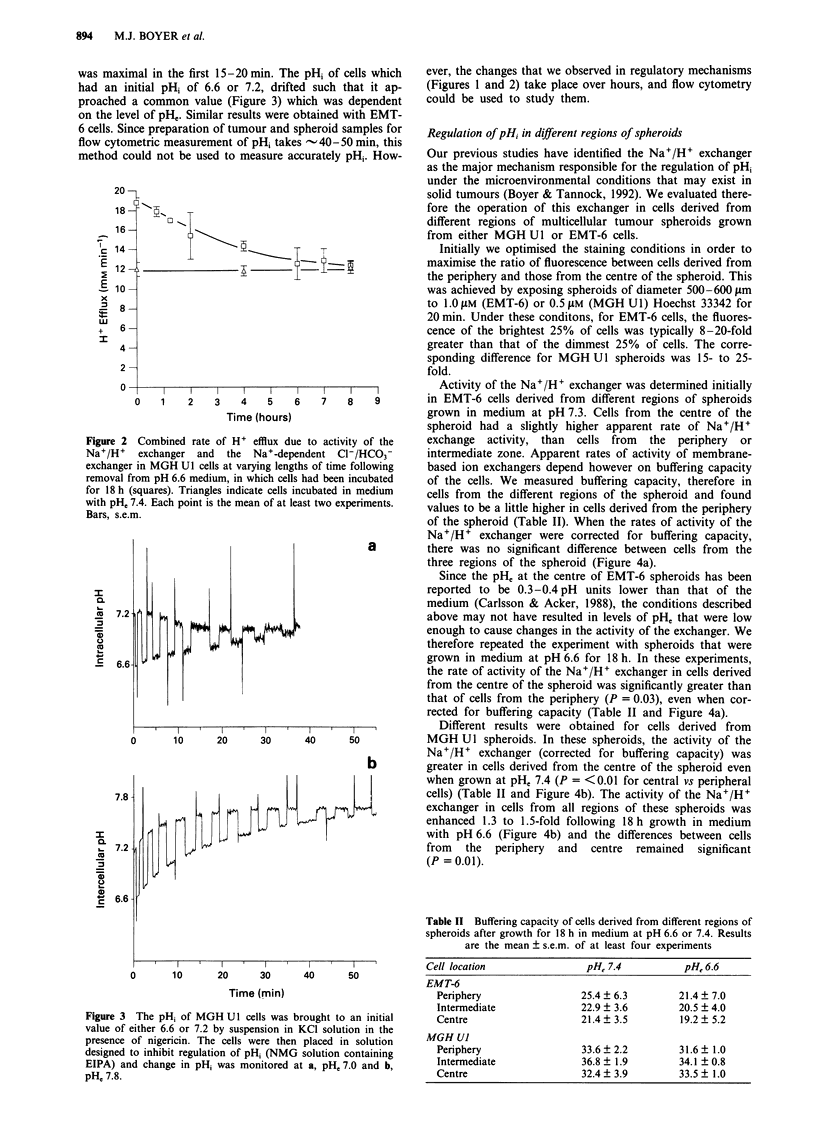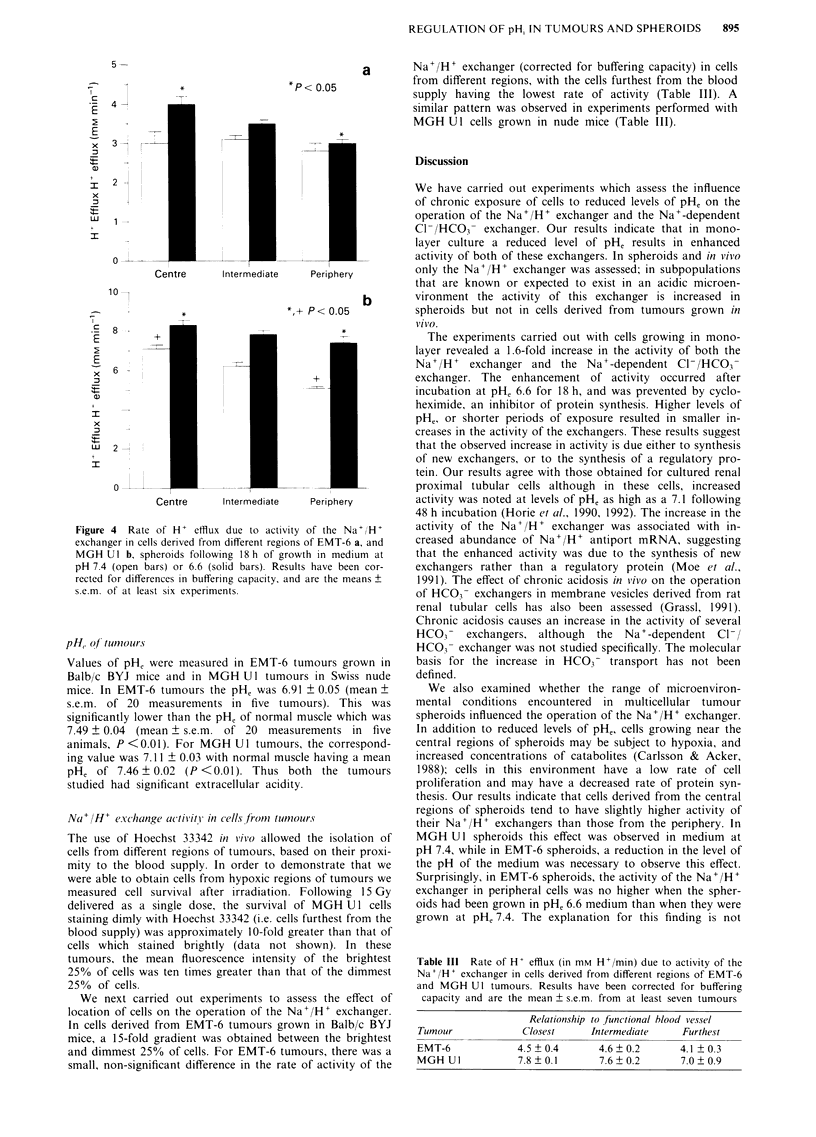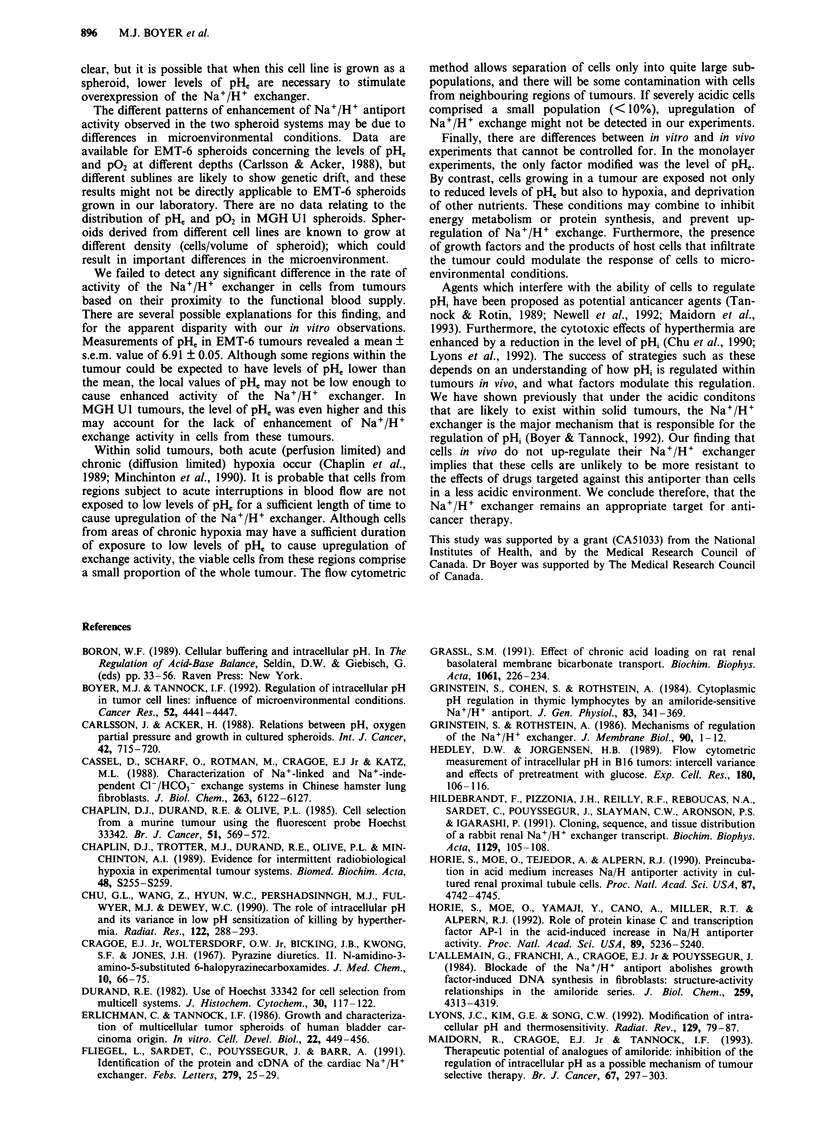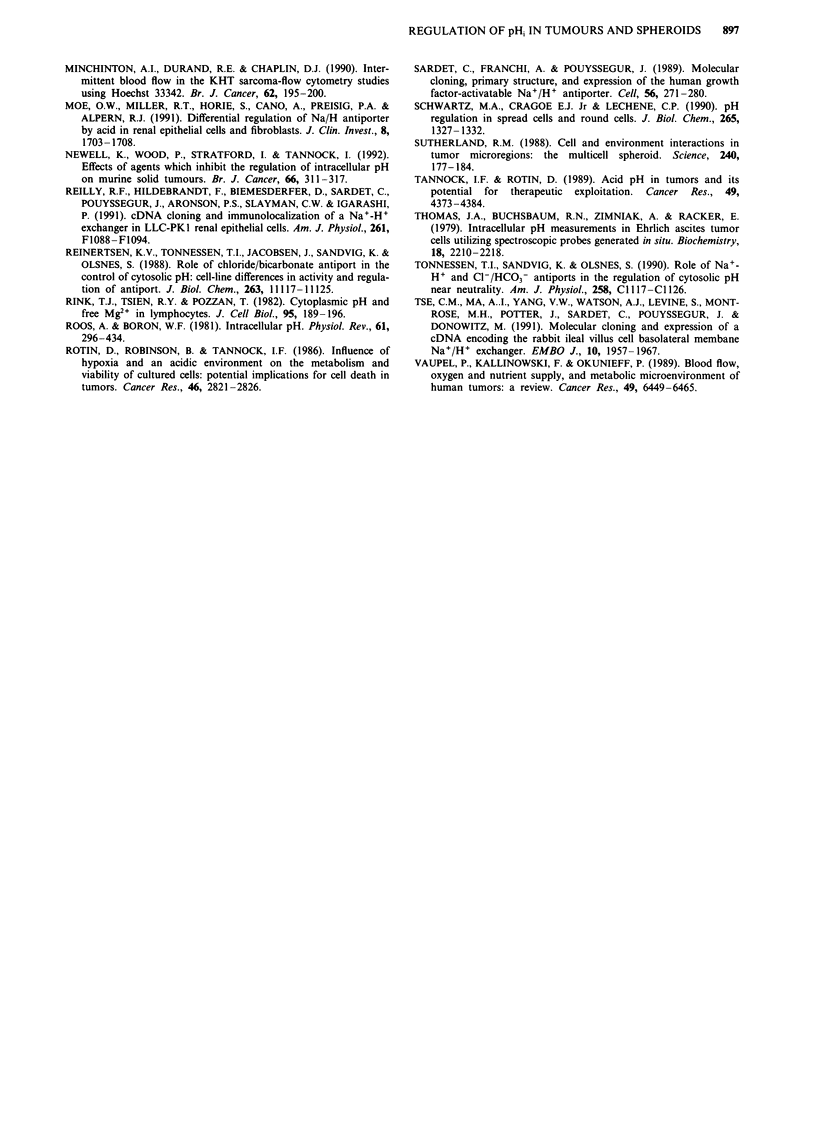# Regulation of intracellular pH in subpopulations of cells derived from spheroids and solid tumours.

**DOI:** 10.1038/bjc.1993.451

**Published:** 1993-11

**Authors:** M. J. Boyer, M. Barnard, D. W. Hedley, I. F. Tannock

**Affiliations:** Department of Medicine, Ontario Cancer Institute, Toronto, Canada.

## Abstract

Solid tumours are known to develop regions of extracellular acidity and survival of tumour cells in such regions depends on membrane-based mechanisms which regulate intracellular pH (pHi). We have therefore developed a method, based on dual staining of cells and flow cytometry, to study the regulation of pHi in subpopulations of tumours and spheroids. The activity of membrane-based pHi regulating transporters was studied in EMT-6 and MGH U1 cells grown in monolayer culture, spheroids, and tumours. pHi was measured with the fluorescent pH probe 2'7'-bis-(2-carboxyethyl)-5-(and-6)carboxyfluorescein, and Hoechst 33342 was used to identify cells from different regions of tumours and spheroids. In monolayer culture, incubation of cells for 18 h at pHe 6.6 led to a 1.3-1.5-fold enhancement in the activity of both the Na+/H+ exchanger and the Na(+)-dependent Cl-@HCO3- exchanger. This effect was prevented by the protein synthesis inhibitor cycloheximide. Cells from the centre of EMT-6 spheroids had increased activity of the Na+/H+ exchanger compared to cells from the periphery, when spheroids were grown in medium at pH 6.6, but not at 7.4. By contrast, in MGH U1 spheroids, cells from the centre had increased activity of the Na+/H+ antiport under both sets of conditions. There was no significant difference in the activity of the Na+/H+ exchanger in cells derived from different subpopulations of EMT-6 tumours or MGH U1 xenografts in nude mice. Although upregulation of Na+/H+ exchange occurs after exposure to acidic conditions in vitro, the microenvironmental conditions found within solid tumours do not appear to cause this effect. Our results suggest the feasibility of pharmacological inhibition of Na+/H+ exchange activity as an approach to therapy directed against nutrient-deprived tumour cells.


### Regulation of intracellular pH in subpopulations of cells derived from spheroids and solid tumours

M.J. Boyer[1], M. Barnard, D.W. Hedley & I.F. Tannock

*Departments of Medicine and Medical Biophysics, Ontario Cancer Institute and University of Toronto, 500 Sherbourne St, Toronto, Ontario, Canada M4X 1K9.*

**Summary** Solid tumours are known to develop regions of extracellular acidity and survival of tumour cells in such regions depends on membrane-based mechanisms which regulate intracellular pH ($pH_i$). We have therefore developed a method, based on dual staining of cells and flow cytometry, to study the regulation of $pH_i$ in subpopulations of tumours and spheroids. The activity of membrane-based $pH_i$ regulating transporters was studied in EMT-6 and MGH U1 cells grown in monolayer culture, spheroids, and tumours. $pH_i$ was measured with the fluorescent pH probe 2'7'-bis-(2-carboxyethyl)-5-(and-6)carboxyfluorescein, and Hoechst 33342 was used to identify cells from different regions of tumours and spheroids. In monolayer culture, incubation of cells for 18 h at $pH_e$ 6.6 led to a 1.3-1.5-fold enhancement in the activity of both the $Na^+/H^+$ exchanger and the $Na^+$-dependent $Cl^-//HCO_3^-$ exchanger. This effect was prevented by the protein synthesis inhibitor cycloheximide. Cells from the centre of EMT-6 spheroids had increased activity of the $Na^+/H^+$ exchanger compared to cells from the periphery, when spheroids were grown in medium at pH 6.6, but not at 7.4. By contrast, in MGH U1 spheroids, cells from the centre had increased activity of the $Na^+/H^+$ antiport under both sets of conditions. There was no significant difference in the activity of the $Na^+/H^+$ exchanger in cells derived from different subpopulations of EMT-6 tumours or MGH U1 xenografts in nude mice. Although upregulation of $Na^+/H^+$ exchange occurs after exposure to acidic conditions *in vitro*, the microenvironmental conditions found within solid tumours do not appear to cause this effect. Our results suggest the feasibility of pharmacological inhibition of $Na^+/H^+$ exchange activity as an approach to therapy directed against nutrient-deprived tumour cells.

Spontaneous cell death has been observed to occur commonly within regions of solid tumours. Although the causes of spontaneous cell death within tumours are not known, the poorly developed vasculature may contribute to this process, by failing to provide adequate nutrients or to remove catabolites (Vaupel *et al.*, 1989). As a result, tumours may contain regions of hypoxia and reduced extracellular pH ($pH_e$) and this combination may be responsible, in part, for the cell death which occurs (Rotin *et al.*, 1986). Although microelectrode measurements have revealed consistently that the average $pH_e$ within solid tumours is approximately 0.5 pH units lower than that in normal tissues, techniques that measure predominantly intracellular pH ($pH_i$), such as $^{31}P$ nuclear magnetic resonance spectroscopy, suggest similar values of $pH_i$ in tumours and normal tissues (Vaupel *et al.*, 1989). These observations imply that tumours cells are able to regulate $pH_i$ under the acidic conditions encountered within solid tumours.

Three major mechanisms allow cells to regulate their $pH_i$ under acidic conditions. These are the buffering capacity of the cytosolic and organellar contents, and two membrane-based transport systems, the $Na^+/H^+$ exchanger and the $Na^+$-dependent $Cl^-/HCO_3^-$ exchanger. The buffering capacity of a cell is its ability to buffer a change in $pH_i$ following the addition (or removal) of $H^+$, and is comprised of both bicarbonate-dependent and non-bicarbonate (mainly protein) components (Roos & Boron, 1981; Boron, 1989). Intracellular buffering provides substantial protection for the cell against effects of an acid load, with most cells capable of buffering millimolar concentrations of $H^+$ (compared to the micromolar concentrations that are normally present) (Roos & Boron, 1981).

The $Na^+/H^+$ exchanger is a membrane based transport mechanism that is ubiquitous in mammalian cells. The exchanger is a 110 kD protein whose gene has been cloned from several tissues in different species (Sardet *et al.*, 1989; Fliegel *et al.*, 1991; Hildebrandt *et al.*, 1991; Reilly *et al.*, 1991; Tse *et al.*, 1991). It uses the inwardly directed $Na^+$ gradient to pump $H^+$ out of cells, and its operation is inhibited by amiloride and its analogs (L'Allemain *et al.*, 1984; Grinstein & Rothstein, 1986). Chronic exposure of cultured renal cells to acidic extracellular conditions has been shown to increase the activity of the $Na^+/H^+$ exchanger by causing the synthesis of new exchange proteins in a process that is dependent on protein kinase C and inhibited by cycloheximide (Horie *et al.*, 1990; Horie *et al.*, 1992).

The other major transporter that regulates $pH_i$ is the $Na^+$-dependent $HCO_3^-/Cl^-$ exchanger. This exchanger has been detected in most but not all cell lines tested (Reinertsen *et al.*, 1988; Tonnessen *et al.*, 1990). It employs the inwardly directed $Na^+$ gradient to exchange intracellular $Cl^-$ for extracellular $HCO_3^-$, and is inhibited by the stilbene derivative 4-4'-diisothiocyanostilbene-2-2'-disulfonic acid (DIDS) (Cassel *et al.*, 1988; Reinertsen *et al.*, 1988). Little is known of its structure and molecular biology. Although chronic acidosis *in vivo* has been shown to increase the activity of renal $HCO_3^-$ exchangers, the mechanisms have not been characterised (Grassl, 1991).

Previous studies in tissue culture have suggestged that under the microenvironmental conditions that exist within the acidic regions of solid tumours, the $Na^+/H^+$ exchanger is likely to be responsible for the majority of regulation of $pH_i$ (Boyer & Tannock, 1992). Agents that inhibit the operation of this exchanger show considerable potential for causing $pH_e$-dependent cyotoxicity and thus selective killing cells in the acidic regions of solid tumours (Tannock & Rotin, 1989; Newell *et al.*, 1992; Maidorn *et al.*, 1993). Rational development of these agents requires an understanding of how regulation of $pH_i$ by tumour cells may be modified by the acidic milieu of tumours. We have examined therefore the operation of the $Na^+/H^+$ exchanger in tumour cells under chronic acidic conditions in culture, and have developed a flow cytometric method to extend these observations to the study of $pH_i$ regulation in different subpopulations of spheroids and experimental tumours.

Correspondence: I.F. Tannock.
[1]Present address: Department of Medical Oncology, Royal Prince Alfred Hospital, Missenden Road, Camperdown, N.S.W. 2050, Australia.
Received 19 March 1993; and in revised form 5 July 1993.

### Materials and methods

### *Cells*

Experiments were performed with murine EMT-6 cells (obtained from Dr R. Sutherland, University of Rochester, NY, USA), and the human bladder carcinoma cell line MGH U1 (obtained from Dr G. Prout, Massachusetts General Hospital, Boston, MA, USA). Cells were maintained routinely in α medium with 5% foetal calf serum (FCS), and new cultures, free of *Mycoplasma*, were re-established from frozen stock every 3 months.

### *Reagents*

Ethylisopropyl-amiloride (EIPA) was synthesised by Aldrich (Milwaukee, WI, USA), as described previously (Cragoe *et al.*, 1967). Hoechst 33342 was obtained from Aldrich (Milwaukee, WI, USA). DIDS was purchased from ICN Biomedicals (St Laurent, PQ, Canada). 2′7′-bis-(2-carboxyethyl)-5-(and-6)carboxyfluorescein (BCECF) acetoxymethyl ester was purchased from Molecular Probes (Eugene, OR, USA). All other reagents were obtained from Sigma (St Louis, MO, USA).

### *Solutions*

Unless otherwise indicated, all solutions were nominally $HCO_3^-$ free. NaCl solution contained 140 mM NaCl, 5 mM KCl, 5 mM glucose, 1 mM $CaCl_2$, 1 mM $MgCl_2$, buffered to the indicated pH with 20 mM MES/Tris. $NaHCO_3$ solution contained 25 mM $NaHCO_3$, 115 mM NaCl, and other components identical to those in NaCl solution. All solutions containing $NaHCO_3$ were prepared in advance, but without the $NaHCO_3$, this was added immediately before use. N-Methyl-D-glucamine (NMG) and KCl solutions were prepared by iso-osmotic replacement of NaCl with NMG and KCl respectively; the other components were identical to those described above for NaCl solution.

pH medium was prepared by adding 20 mM MES to regular medium and adjusting to the desired pH with HCl or NaOH. Medium containing $HCO_3^-$ was adjusted to the desired pH by using HCl or NaOH. Prior to use, it was bubbled with 5% $CO_2$ for 2 h and the pH was then re-adjusted. By following this procedure, the pH of medium remained within 0.1 pH units of the desired value for up to 24 h.

### *Evaluation of $pH_i$ and its regulation for cells in monolayer*

Cells grown as a monolayer on glass coverslips were exposed to 2 μg $ml^{-1}$ of the acetoxymethyl ester of BCECF in serum free α-medium at 37°C for 20 min. The coverslip was then placed into a cuvette using a specially designed holder aligned at an angle of 30° to the excitation beam of a Perkin Elmer LS3 fluorometer (Perkin Elmer, Mississauga, Ontario). The holder also served as a cap for the cuvette, minimising the loss of $CO_2$. The cuvette was equipped with a perfusion system to allow exchange of the buffer surrounding the cells. Exchanges were made with a volume of buffer at least ten times greater than the volume contained within the cuvette. The temperature of the solution in the cuvette was controlled precisely and all experiments were carried out at 37°C.

Within the range of $pH_i$ 6.0–7.5, fluorescence intensity of BCECF at 525 nM (following excitation at 495 nM) is linearly related to $pH_i$ (Rink *et al.*, 1982). At three time points during each experiment (prior to intracellular acidification, following intracellular acidification, and at the end of the experiment), fluorescence intensity was measured at the same emission wavelength but with an excitation wavelength of 440 nM (Schwartz *et al.*, 1990). Following excitation at this wavelength, fluorescence intensity is independent of $pH_i$, and depends only on the amount of BCECF present. The ratio of fluorescence at pH-dependent and independent wavelengths provides therefore an estimate of $pH_i$ that is independent of the amount of BCECF present in cells or of loss of cells from coverslips. Calibration of fluorescence measurements was performed using the ionophore nigericin, in a solution containing 140 mM $K^+$, as described previously (Thomas *et al.*, 1979).

Intracellular acidification was achieved by placing cells in KCl solution containing $NH_4Cl$ for 30 min. Acidification to a level determined by the concentration of $NH_4^+$ used, was then produced by exchanging the $NH_4Cl$ containing solution with a $NH_4Cl$-free solution (Boron, 1989).

Cytoplasmic acidification was carried out in $Na^+$ and $HCO_3^-$-free buffer. In experiments designed to measure the activity of the $Na^+/H^+$ exchanger, this buffer was replaced after intracellular acidification by $Na^+$-containing, $HCO_3^-$-free buffer, pH 7.4. By contrast, in those experiments where the action of the $Na^+$-dependent $Cl^-/HCO_3^-$ exchanger was investigated, the buffer was replaced by $Na^+$ and $HCO_3^-$-containing buffer, pH 7.4, with 10 μM EIPA (which provides inhibition of $Na^+/H^+$ exchange activity). The combined activity of both exchangers was evaluated by exchanging with $Na^+$ and $HCO_3^-$-containing buffer, pH 7.4, in the absence of EIPA. Following the change in extracellular buffer, the maximal rate of the resulting intracellular alkalinisation was measured by the fluorometre. The results of these experiments were converted into $H^+$ efflux by multiplying the observed rates of change of $pH_i$ by the total buffering capacity of the cells (see below).

In some experiments, we assessed the influence of chronic exposure to low levels of $pH_e$ on the activity of the $pH_i$ regulating exchangers. For these experiments, cells growing on glass coverslips were placed into pH adjusted medium at varying times prior to the evaluation of the membrane-based exchangers which regulate $pH_i$. Measurement of the activity of the exchangers was carried out in buffers with pH 7.4.

### *Calculation of intracellular buffering capacity*

Buffering capacity is the capacity of a cell to buffer changes in $pH_i$ following addition or removal of $H^+$; it is defined as the ratio of moles of $H^+$ (or $OH^-$) added to the resulting change of $pH_i$ i.e. $\Delta[H^+]/\Delta pH_i$ (Roos & Boron, 1981). In order to measure intrinsic (non-bicarbonate) intracellular buffering capacity, cells were exposed to $HCO_3^-$-free NMG solution containing 3 mM ammonium chloride for 5 min, followed by replacement of the extracellular fluid with $NH_4$-free NMG solution. The resulting fall in $pH_i$ was measured and used to calculate intrinsic buffering capacity using the formula described previously (Boyer & Tannock, 1992). Measurements of intrinsic buffering capacity were made at the resting level of $pH_i$ only, since values have been shown to be constant over the range of $pH_i$ 6.4–7.2 (Grinstein *et al.*, 1984). Bicarbonate buffering capacity was calculated as 2.3 $[HCO_3^-]_i$ (Boron, 1989). The value of $[HCO_3^-]_i$ was calculated from knowledge of $pH_i$, $pH_e$ and $[HCO_3^-]_e$. The total buffering capacity is the sum of intrinsic buffering capacity and bicarbonate buffering capacity.

### *Spheroids*

Some experiments were performed with multicellular tumour spheroids. Spheroids provide a tissue culture model for tumours where cells exist within a variable microenvironment (Sutherland, 1988). MGH U1 spheroids were grown from a subline of MGH U1 cells, as described previously (Erlichman & Tannock, 1986). They were maintained routinely in spinner flasks containing HEPES-buffered, $HCO_3^-$-free medium supplemented with 10% FCS. EMT-6 spheroids were grown by seeding EMT-6 cells into uncoated Petri dishes, and allowing them to grow overnight as described previously (Newell *et al.*, 1992). The following day, the small spheroids that had formed were placed into spinner flasks with HEPES-buffered, $HCO_3^-$-free medium and 15% FCS. Medium was exchanged daily thereafter.

Subpopulations of cells at different depths in spheroids were obtained by exposure of spheroids to the fluorescent dye Hoechst 33342 followed by dissociation and flow cytometry (Durand, 1982). Spheroids (diameter 500–600 μm) growing in a spinner flask were exposed for 20 min to Hoechst 33342 (0.5 or 1 μM for EMT-6 and MGH U1 spheroids respectively). They were then rinsed three times in ice cold PBS and dissociated using a combination of trypsin and gentle mechanical disaggregation.

### *Growth of tumours*

MGH U1 bladder carcinoma and EMT-6 mammary sarcoma were grown in inbred female Swiss Nude (Taconic; Germantown, NY, USA) and Balb/c BYJ (Jackson Laboratories; Bar Harbor, Maine, USA) mice respectively. Tumours were initiated by injecting $2.5-5 \times 10^5$ cells into the left hind leg. Growth of the tumours was monitored by passing the leg through a strip of lucite with graded circular holes. The diameter of the tumour-bearing leg was converted to an estimate of tumour weight using a previously defined calibration curve. Tumours were used for experiments when they had attained a weight of 0.3–0.5 g (approximately 9 days of growth).

Subpopulations of tumour cells at different distances from functional blood vessels were obtained by administration of Hoechst 33342, followed by tumour excision, dissociation and flow cytometry (Chaplin *et al.*, 1985). In Balb/c mice a 30 min infusion of Hoechst 33342 (1 ml of a 1 mg $ml^{-1}$ solution in water) was administered via the lateral tail vein. We were unable to perform tail vein infusions consistently in nude mice and in these animals an intraperitoneal injection of 1.5 mg of Hoechst 33342 in 0.75 ml of water was used. Thirty minutes after injection (or at the conclusion of the infusion) mice were killed by cervical dislocation and the tumour excised. The tumour was placed into ice cold PBS and minced into fine pieces using crossed scalpel blades. A single cell suspension was produced by treatment with trypsin and DNAase 1 for 10–15 min (at 37°C) followed by passage through a fine screen.

### *Potential for evaluation of $pH_i$ from dissociated spheroids and tumours*

We wished to adapt a flow cytometric method to measure $pH_i$ in cells from subpopulations of spheroids and tumours (Hedley & Jorgensen, 1989). Initially cultured EMT-6 and MGH U1 cells were used to model the changes in $pH_i$ that might occur during sample preparation. Following trypsinisation, cells were suspended in serum-free α-medium and exposed to BCECF-AM, 2 μg $ml^{-1}$ for 20 min, at 37°C. In order to set the level of $pH_i$ to either 6.6 or 7.2, the cells were then centrifuged and resuspended in KCl solution at $pH_e$ 6.6 or 7.2, which contained nigericin, 2 mg $ml^{-1}$. After 5 min, the cells were centrifuged once more, and resuspended in room-temperature NMG buffer containing 10 μM EIPA at various levels of $pH_e$; this solution provides complete inhibition of $Na^+/H^+$ and $Na^+$-dependent $HCO_3^-/Cl^-$ exchangers that regulate $pH_i$ under acidic conditions. The cell suspensions were then placed into two cuvettes in an Aminco-Bowman Series 2 Fluorometer (SLM, Urbana, IL, USA); this machine allows rapid alternation of fluorescence measurements from two cuvettes and $pH_i$ was monitored, using the ratio method described earlier, by alternating between each of the cuvettes for up to 60 min.

### *Evaluation of $pH_i$ and its regulation in dissociated spheroids and tumours*

Regulation of $pH_i$ in cells derived from spheroids or tumours was assessed by flow cytometry. The instrument used was a Coulter Epics Elite flow cytometer (Coulter Electronics, Hialeah, FL, USA), equipped with air cooled HeCd (325 nm) and argon (488 nm) lasers, and modified to allow constant control of sample temperature via a circulating water bath. The level of $pH_i$ was measured by the ratio of the 525 nm (pH dependent) and 640 nm (pH independent) emissions of BCECF following excitation at 488 nm. Calibration of fluorescence ratio measurements was performed using the ionophore nigericin, in a solution containing 140 mM $K^+$, as described previously (Thomas *et al.*, 1979). Hoechst fluorescence was measured at 450 nm following excitation at 325 nm.

A single cell suspension was prepared from tumours or spheroids as described above. After exposure to the 2 μg $ml^{-1}$ of the acetoxymethyl ester of BCECF in serum-free α-medium for 20 min at 37°C, cells were resuspended in NMG buffer. Intracellular acidification was produced by exposure to nigericin for 3–4 min (spheroids) or by exposure to and subsequent removal of 10 mM $NH_4^+$ (tumours); cells were then centrifuged. There was no difference in the acidification produced, or subsequent activity of the $Na^+/H^+$ exchanger following use of the two different methods of acidification (data not shown). Recovery from acidification was measured following resuspension of the pellet in sodium buffer.

Flow cytometric measurements of intrinsic buffering capacity were made in a manner analogous to that described above for cells growing in monolayer. However, rather han measuring the fall in $pH_i$ following removal of $NH_4^+$, the immediate increase in $pH_i$ resulting from exposure of cells to 5 mM $NH_4^+$ was determined. The measurements and calculations were otherwise identical.

Regulation of $pH_i$ in different spheroid and tumour subpopulations was assessed by cell separation based on the intensity of Hoechst staining of the cells (Durand, 1982; Chaplin *et al.*, 1985). In our experiments, cells were divided into three populations, representing the brightest 25% of cells, the dimmest 25% of cells, and the intermediate 50%. The rate of change of $pH_i$ after addition of sodium was then determined for each of these subpopulations, by calculating the average $pH_i$ over 1 min intervals, for 8–10 min. The mean values of different subpopulations were compared using Student's *t*-test.

### *Identification and gating of tumour cells*

Because tumours contain a variety of host and stromal cells it was necessary to identify the tumour cells prior to measurement of $pH_i$. This was achieved by gating the sample on the basis of forward and 90° light scatter which provides an indirect measure of cell size. Since the tumour cells were larger than the normal host cells these measurements could be used to identify tumour cells. To assess the effectiveness of this gating procedure we measured also the ploidy of the cells; both the EMT-6 and MGH U1 tumours are aneuploid while the host cells are diploid. A parallel sample was stained with 10 μM Hoechst 33342 for 30 min, to obtain a DNA histogram. In the ungated population of cells, typically 40–60% of the cells were aneuploid. Following gating as described above, 85–90% of the cells were aneuploid, and only these cells were included in the measurement of $pH_i$. The excluded population contained 5–10% aneuploid cells.

### *Irradiation and sorting of tumours*

In order to ensure that our separation procedure resulted in selection of cells from hypoxic (and presumably acidic) regions of tumours, we assessed the survival of different tumour subpopulations following irradiation *in vivo*. Immediately after intraperitoneal injection of Hoechst 33342, unanesthetised and unrestrained tumour bearing mice were irradiated with a dose of 15 Gy using a $^{137}Cs$ source at a dose rate of 0.58 Gy $min^{-1}$. Following this, the mice were killed, and the tumours excised and prepared for flow cytometry as described above. Cells were sorted on the basis of fluorescence intensity of Hoechst 33342 into three populations identical to those in the $pH_i$ regulation experiments described above. The sorted cells were incubated in culture dishes containing α-medium, in a humidified atmosphere containing 5% $CO_2$ at 37°C for 12 days. The number of colonies containing >50 cells was then counted. The surviving fraction was expressed relative to that of unirradiated controls.

*Measurement of $pH_e$ in vivo*

Mice were anesthetised with sodium pentobarbital (M.T.C. Pharmaceuticals; Cambridge, Ontario), 65 mg kg$^{-1}$ body weight. Measurements of $pH_e$ were made using a miniature glass electrode which has a sensing area of diameter 500 $\mu$m (model MI-408b, Microelectrodes Inc; New Hampshire) against a silver–silver chloride reference electrode (model MI-402, Microelectrodes Inc; New Hampshire) using a high impedance pH meter (PHM 82, Radiometer, Copenhagen). The reference electrode was inserted subcutaneously on the back, and bathed in phosphate buffered saline. The pH microelectrode was inserted directly into the tumour or muscle tissue after the overlying skin had been removed. Measurements of tumour $pH_e$ were made at increments of 50–75 $\mu$m along a single track at a depth of 200–500 $\mu$m into the tumour using a specially designed micromanipulator. At least four measurements were made per tumour.

### Results

*Effects of low $pH_e$ exposure on regulation of $pH_i$*

Initial experiments were undertaken to determine whether the activity of the $Na^+/H^+$ exchanger and the $Na^+$-dependent $Cl^-/HCO_3^-$ exchanger were altered by growth of cells under acidic conditions. Cells were grown for up to 18 h at levels of $pH_e$ in the range 6.6 to 7.2. Under these conditions, clonogenic survival of the cells was not altered, and cells grew normally (data not shown). There was a time and $pH_e$ dependent increase in the combined activity of the $Na^+/H^+$ exchanger and the $Na^+$-dependent $Cl^-/HCO_3^-$ exchanger in MGH U1 cells (Figure 1). The maximum increase, a 1.6-fold enhancement of activity, occurred after 18 h incubation at $pH_e$ 6.6; evaluation after longer times was not undertaken because of loss of cellular viability. Similar results were obtained with EMT-6 cells (Table I).

The apparent increase in the combined activity of the $Na^+/H^+$ antiport and the $Na^+$-dependent $Cl^-/HCO_3^-$ exchanger could have been due to changes in the intrinsic buffer capacity of the cell after low $pH_e$ exposure. We therefore measured intrinsic buffering capacity after MGH U1 cells had been growing at $pH_e$ 6.6 for 18 h. There was no difference between these values (38.7 ± 4.0 mM $H^+$/pH unit (mean ± s.e.m of four experiments)) and control values (39.3 ± 3.8 mM $H^+$/pH unit). This result implies that

**Figure 1** Combined rate of $H^+$ efflux due to activity of the $Na^+/H^+$ exchanger and the $Na^+$-dependent $Cl^-/HCO_3^-$ exchanger in MGH U1 cells after incubation at the pH indicated for 1 (diamonds), 6 (triangles) or 18 (open squares) hours. Rates are also shown for 18 h exposure in the presence of 3 $\mu$g ml$^{-1}$ cycloheximide (solid squares). Results have been corrected for differences in buffering capacity and are expressed as a percentage of the rate in control cells incubated at pH 7.4. Each point represents the mean of at least three experiments. Bars, s.e.m.

**Table I** Rates of $H^+$ efflux (in mM $H^+$/min) in EMT-6 and MGH U1 cells after 18 h incubation at the level of $pH_e$ indicated. Results have been corrected for differences in buffering capacity, and are the mean ± s.e.m. of at least three experiments

| | *Incubation pH* | |
|---|---|---|
| *EMT-6* | *$pH_e$ 7.4* | *$pH_e$ 6.6* |
| $Na^+/H^+$ exchanger | 3.2 ± 0.1 | 4.7 ± 0.2 |
| $Na^+$-dependent $Cl^-/HCO_3^-$ exchanger | 2.0 ± 0.2 | 3.1 ± 0.2 |
| Both combined | 4.9 ± 0.5 | 7.7 ± 1.3 |
| *MGH U1* | | |
| $Na^+/H^+$ exchanger | 5.8 ± 0.8 | 8.9 ± 0.4 |
| $Na^+$-dependent $Cl^-/HCO_3^-$ exchanger | 6.4 ± 0.3 | 9.4 ± 0.4 |
| Both combined | 12.0 ± 0.8 | 19.4 ± 0.2 |

the observed changes were due to alterations in either the number or activity of the exchangers.

The observed increase in the combined activity of the $Na^+/H^+$ exchanger and the $Na^+$-dependent $Cl^-/HCO_3^-$ exchanger may have been due to an increase in the activity of both of the exchangers or only one of them. We therefore repeated the experiments in the absence of $HCO_3^-$ (to measure activity of the $Na^+/H^+$ exchanger) or in the presence of EIPA (to measure activity of the $Na^+$-dependent $Cl^-/HCO_3^-$ exchanger). Table I shows that the rate of $pH_i$ recovery due to each of the two exchangers was increased by acid incubation.

In order to determine whether the increase in the activity of the exchangers was dependent on protein synthesis, experiments were carried out in the presence of the protein synthesis inhibitor, cycloheximide. Exposure of EMT-6 or MGH U1 cells in the presence of up to 10 $\mu$g ml$^{-1}$ of cycloheximide for 18 h did not result in any decrease in clonogenic survival at either $pH_e$ 6.6 or 7.4 although growth of the cultures was inhibited (data not shown). After 18 h incubation at $pH_e$ 6.6 in the presence of cycloheximide (3 $\mu$g ml$^{-1}$) there was no increase in the combined activity of the $Na^+/H^+$ exchanger and the $Na^+$-dependent $HCO_3^-$ exchanger in MGH U1 cells when compared to controls incubated at $pH_e$ 7.4 in the absence of drug (Figure 1). This result is not influenced by possible differences in the number of cells in the presence or absence of cycloheximide, since $pH_i$ was determined as a ratio of fluorescence at pH-sensitive and insensitive wavelengths. The result implies that increased capacity for regulation of $pH_i$ after chronic exposure to acidic conditions is dependent on protein synthesis. Similar results were obtained for EMT-6 cells (data not shown).

We also evaluated the time taken for the activity of the exchangers to return to normal after cells were placed in $pH_e$ 7.4 medium following an 18 h incubation at $pH_e$ 6.6. In MGH U1 cells, the combined activity of the $Na^+/H^+$ antiport and the $Na^+$-dependent $HCO_3^-/Cl^-$ exchanger returned to control values over 8 h (Figure 2).

*Potential of flow cytometry to measure $pH_i$ in subpopulations of spheroids and tumours*

A flow-cytometric method for the measurement of $pH_i$ in cells derived from dissociated tumours has been described (Hedley & Jorgensen, 1989). This technique is based on dissociation of the tumour into cold, bicarbonate-free buffer, containing amiloride, in order to prevent changes in $pH_i$ during preparation of a single cell suspension for flow cytometry; any change in the level of $pH_i$ during processing of the sample would lead to inaccuracy of the measured values.

In order to adapt this method for the measurement of $pH_i$ in cells from subpopulations of spheroids and tumours, we first modelled the changes in $pH_i$ that take place during sample preparation. Experiments were carried out at room temperature because of condensation on the cuvette at 4°C. There was considerable drift of the level of $pH_i$ in MGH U1 cells suspended in NMG buffer containing EIPA. This drift was maximal in the first 15–20 min. The $pH_i$ of cells which had an initial $pH_i$ of 6.6 or 7.2, drifted such that it approached a common value (Figure 3) which was dependent on the level of $pH_e$. Similar results were obtained with EMT-6 cells. Since preparation of tumour and spheroid samples for flow cytometric measurement of $pH_i$ takes ~40–50 min, this method could not be used to measure accurately $pH_i$. However, the changes that we observed in regulatory mechanisms (Figures 1 and 2) take place over hours, and flow cytometry could be used to study them.

**Figure 2** Combined rate of $H^+$ efflux due to activity of the $Na^+/H^+$ exchanger and the $Na^+$-dependent $Cl^-/HCO_3^-$ exchanger in MGH U1 cells at varying lengths of time following removal from pH 6.6 medium, in which cells had been incubated for 18 h (squares). Triangles indicate cells incubated in medium with $pH_e$ 7.4. Each point is the mean of at least two experiments. Bars, s.e.m.

**Figure 3** The $pH_i$ of MGH U1 cells was brought to an initial value of either 6.6 or 7.2 by suspension in KCl solution in the presence of nigericin. The cells were then placed in solution designed to inhibit regulation of $pH_i$ (NMG solution containing EIPA) and change in $pH_i$ was monitored at **a**, $pH_e$ 7.0 and **b**, $pH_e$ 7.8.

*Regulation of $pH_i$ in different regions of spheroids*

Our previous studies have identified the $Na^+/H^+$ exchanger as the major mechanism responsible for the regulation of $pH_i$ under the microenvironmental conditions that may exist in solid tumours (Boyer & Tannock, 1992). We evaluated therefore the operation of this exchanger in cells derived from different regions of multicellular tumour spheroids grown from either MGH U1 or EMT-6 cells.

Initially we optimised the staining conditions in order to maximise the ratio of fluorescence between cells derived from the periphery and those from the centre of the spheroid. This was achieved by exposing spheroids of diameter 500–600 μm to 1.0 μM (EMT-6) or 0.5 μM (MGH U1) Hoechst 33342 for 20 min. Under these conditons, for EMT-6 cells, the fluorescence of the brightest 25% of cells was typically 8–20-fold greater than that of the dimmest 25% of cells. The corresponding difference for MGH U1 spheroids was 15- to 25-fold.

Activity of the $Na^+/H^+$ exchanger was determined initially in EMT-6 cells derived from different regions of spheroids grown in medium at pH 7.3. Cells from the centre of the spheroid had a slightly higher apparent rate of $Na^+/H^+$ exchange activity, than cells from the periphery or intermediate zone. Apparent rates of activity of membrane-based ion exchangers depend however on buffering capacity of the cells. We measured buffering capacity, therefore in cells from the different regions of the spheroid and found values to be a little higher in cells derived from the periphery of the spheroid (Table II). When the rates of activity of the $Na^+/H^+$ exchanger were corrected for buffering capacity, there was no significant difference between cells from the three regions of the spheroid (Figure 4a).

Since the $pH_e$ at the centre of EMT-6 spheroids has been reported to be 0.3–0.4 pH units lower than that of the medium (Carlsson & Acker, 1988), the conditions described above may not have resulted in levels of $pH_e$ that were low enough to cause changes in the activity of the exchanger. We therefore repeated the experiment with spheroids that were grown in medium at pH 6.6 for 18 h. In these experiments, the rate of activity of the $Na^+/H^+$ exchanger in cells derived from the centre of the spheroid was significantly greater than that of cells from the periphery ($P = 0.03$), even when corrected for buffering capacity (Table II and Figure 4a).

Different results were obtained for cells derived from MGH U1 spheroids. In these spheroids, the activity of the $Na^+/H^+$ exchanger (corrected for buffering capacity) was greater in cells derived from the centre of the spheroid even when grown at $pH_e$ 7.4 ($P = <0.01$ for central *vs* peripheral cells) (Table II and Figure 4b). The activity of the $Na^+/H^+$ exchanger in cells from all regions of these spheroids was enhanced 1.3 to 1.5-fold following 18 h growth in medium with pH 6.6 (Figure 4b) and the differences between cells from the periphery and centre remained significant ($P = 0.01$).

**Table II** Buffering capacity of cells derived from different regions of spheroids after growth for 18 h in medium at pH 6.6 or 7.4. Results are the mean ± s.e.m. of at least four experiments

| *Cell location* | *$pH_e$ 7.4* | *$pH_e$ 6.6* |
|---|---|---|
| *EMT-6* | | |
| Periphery | 25.4 ± 6.3 | 21.4 ± 7.0 |
| Intermediate | 22.9 ± 3.6 | 20.5 ± 4.0 |
| Centre | 21.4 ± 3.5 | 19.2 ± 5.2 |
| *MGH U1* | | |
| Periphery | 33.6 ± 2.2 | 31.6 ± 1.0 |
| Intermediate | 36.8 ± 1.9 | 34.1 ± 0.8 |
| Centre | 32.4 ± 3.9 | 33.5 ± 1.0 |

**Figure 4** Rate of $H^+$ efflux due to activity of the $Na^+/H^+$ exchanger in cells derived from different regions of EMT-6 **a**, and MGH U1 **b**, spheroids following 18 h of growth in medium at pH 7.4 (open bars) or 6.6 (solid bars). Results have been corrected for differences in buffering capacity, and are the means ± s.e.m. of at least six experiments.

### *$pH_e$ of tumours*

Values of $pH_e$ were measured in EMT-6 tumours grown in Balb/c BYJ mice and in MGH U1 tumours in Swiss nude mice. In EMT-6 tumours the $pH_e$ was 6.91 ± 0.05 (mean ± s.e.m. of 20 measurements in five tumours). This was significantly lower than the $pH_e$ of normal muscle which was 7.49 ± 0.04 (mean ± s.e.m. of 20 measurements in five animals, $P < 0.01$). For MGH U1 tumours, the corresponding value was 7.11 ± 0.03 with normal muscle having a mean $pH_e$ of 7.46 ± 0.02 ($P < 0.01$). Thus both the tumours studied had significant extracellular acidity.

### *$Na^+/H^+$ exchange activity in cells from tumours*

The use of Hoechst 33342 *in vivo* allowed the isolation of cells from different regions of tumours, based on their proximity to the blood supply. In order to demonstrate that we were able to obtain cells from hypoxic regions of tumours we measured cell survival after irradiation. Following 15 Gy delivered as a single dose, the survival of MGH U1 cells staining dimly with Hoechst 33342 (i.e. cells furthest from the blood supply) was approximately 10-fold greater than that of cells which stained brightly (data not shown). In these tumours, the mean fluorescence intensity of the brightest 25% of cells was ten times greater than that of the dimmest 25% of cells.

We next carried out experiments to assess the effect of location of cells on the operation of the $Na^+/H^+$ exchanger. In cells derived from EMT-6 tumours grown in Balb/c BYJ mice, a 15-fold gradient was obtained between the brightest and dimmest 25% of cells. For EMT-6 tumours, there was a small, non-significant difference in the rate of activity of the $Na^+/H^+$ exchanger (corrected for buffering capacity) in cells from different regions, with the cells furthest from the blood supply having the lowest rate of activity (Table III). A similar pattern was observed in experiments performed with MGH U1 cells grown in nude mice (Table III).

### Discussion

We have carried out experiments which assess the influence of chronic exposure of cells to reduced levels of $pH_e$ on the operation of the $Na^+/H^+$ exchanger and the $Na^+$-dependent $Cl^-/HCO_3^-$ exchanger. Our results indicate that in monolayer culture a reduced level of $pH_e$ results in enhanced activity of both of these exchangers. In spheroids and *in vivo* only the $Na^+/H^+$ exchanger was assessed; in subpopulations that are known or expected to exist in an acidic microenvironment the activity of this exchanger is increased in spheroids but not in cells derived from tumours grown *in vivo*.

The experiments carried out with cells growing in monolayer revealed a 1.6-fold increase in the activity of both the $Na^+/H^+$ exchanger and the $Na^+$-dependent $Cl^-/HCO_3^-$ exchanger. The enhancement of activity occurred after incubation at $pH_e$ 6.6 for 18 h, and was prevented by cycloheximide, an inhibitor of protein synthesis. Higher levels of $pH_e$, or shorter periods of exposure resulted in smaller increases in the activity of the exchangers. These results suggest that the observed increase in activity is due either to synthesis of new exchangers, or to the synthesis of a regulatory protein. Our results agree with those obtained for cultured renal proximal tubular cells although in these cells, increased activity was noted at levels of $pH_e$ as high as a 7.1 following 48 h incubation (Horie *et al.*, 1990, 1992). The increase in the activity of the $Na^+/H^+$ exchanger was associated with increased abundance of $Na^+/H^+$ antiport mRNA, suggesting that the enhanced activity was due to the synthesis of new exchangers rather than a regulatory protein (Moe *et al.*, 1991). The effect of chronic acidosis *in vivo* on the operation of $HCO_3^-$ exchangers in membrane vesicles derived from rat renal tubular cells has also been assessed (Grassl, 1991). Chronic acidosis causes an increase in the activity of several $HCO_3^-$ exchangers, although the $Na^+$-dependent $Cl^-/HCO_3^-$ exchanger was not studied specifically. The molecular basis for the increase in $HCO_3^-$ transport has not been defined.

We also examined whether the range of microenvironmental conditions encountered in multicellular tumour spheroids influenced the operation of the $Na^+/H^+$ exchanger. In addition to reduced levels of $pH_e$, cells growing near the central regions of spheroids may be subject to hypoxia, and increased concentrations of catabolites (Carlsson & Acker, 1988); cells in this environment have a low rate of cell proliferation and may have a decreased rate of protein synthesis. Our results indicate that cells derived from the central regions of spheroids tend to have slightly higher activity of their $Na^+/H^+$ exchangers than those from the periphery. In MGH U1 spheroids this effect was observed in medium at pH 7.4, while in EMT-6 spheroids, a reduction in the level of the pH of the medium was necessary to observe this effect. Surprisingly, in EMT-6 spheroids, the activity of the $Na^+/H^+$ exchanger in peripheral cells was no higher when the spheroids had been grown in $pH_e$ 6.6 medium than when they were grown at $pH_e$ 7.4. The explanation for this finding is not

**Table III** Rate of $H^+$ efflux (in mM $H^+$/min) due to activity of the $Na^+/H^+$ exchanger in cells derived from different regions of EMT-6 and MGH U1 tumours. Results have been corrected for buffering capacity and are the mean ± s.e.m. from at least seven tumours

| *Tumour* | *Relationship to functional blood vessel* Closest | Intermediate | Furthest |
|---|---|---|---|
| EMT-6 | 4.5 ± 0.4 | 4.6 ± 0.2 | 4.1 ± 0.3 |
| MGH U1 | 7.8 ± 0.1 | 7.6 ± 0.2 | 7.0 ± 0.9 |

clear, but it is possible that when this cell line is grown as a spheroid, lower levels of $pH_e$ are necessary to stimulate overexpression of the $Na^+/H^+$ exchanger.

The different patterns of enhancement of $Na^+/H^+$ antiport activity observed in the two spheroid systems may be due to differences in microenvironmental conditions. Data are available for EMT-6 spheroids concerning the levels of $pH_e$ and $pO_2$ at different depths (Carlsson & Acker, 1988), but different sublines are likely to show genetic drift, and these results might not be directly applicable to EMT-6 spheroids grown in our laboratory. There are no data relating to the distribution of $pH_e$ and $pO_2$ in MGH U1 spheroids. Spheroids derived from different cell lines are known to grow at different density (cells/volume of spheroid); which could result in important differences in the microenvironment.

We failed to detect any significant difference in the rate of activity of the $Na^+/H^+$ exchanger in cells from tumours based on their proximity to the functional blood supply. There are several possible explanations for this finding, and for the apparent disparity with our *in vitro* observations. Measurements of $pH_e$ in EMT-6 tumours revealed a mean ± s.e.m. value of 6.91 ± 0.05. Although some regions within the tumour could be expected to have levels of $pH_e$ lower than the mean, the local values of $pH_e$ may not be low enough to cause enhanced activity of the $Na^+/H^+$ exchanger. In MGH U1 tumours, the level of $pH_e$ was even higher and this may account for the lack of enhancement of $Na^+/H^+$ exchange activity in cells from these tumours.

Within solid tumours, both acute (perfusion limited) and chronic (diffusion limited) hypoxia occur (Chaplin *et al.*, 1989; Minchinton *et al.*, 1990). It is probable that cells from regions subject to acute interruptions in blood flow are not exposed to low levels of $pH_e$ for a sufficient length of time to cause upregulation of the $Na^+/H^+$ exchanger. Although cells from areas of chronic hypoxia may have a sufficient duration of exposure to low levels of $pH_e$ to cause upregulation of exchange activity, the viable cells from these regions comprise a small proportion of the whole tumour. The flow cytometric method allows separation of cells only into quite large subpopulations, and there will be some contamination with cells from neighbouring regions of tumours. If severely acidic cells comprised a small population (<10%), upregulation of $Na^+/H^+$ exchange might not be detected in our experiments.

Finally, there are differences between *in vitro* and *in vivo* experiments that cannot be controlled for. In the monolayer experiments, the only factor modified was the level of $pH_e$. By contrast, cells growing in a tumour are exposed not only to reduced levels of $pH_e$ but also to hypoxia, and deprivation of other nutrients. These conditions may combine to inhibit energy metabolism or protein synthesis, and prevent upregulation of $Na^+/H^+$ exchange. Furthermore, the presence of growth factors and the products of host cells that infiltrate the tumour could modulate the response of cells to microenvironmental conditions.

Agents which interfere with the ability of cells to regulate $pH_i$ have been proposed as potential anticancer agents (Tannock & Rotin, 1989; Newell *et al.*, 1992; Maidorn *et al.*, 1993). Furthermore, the cytotoxic effects of hyperthermia are enhanced by a reduction in the level of $pH_i$ (Chu *et al.*, 1990; Lyons *et al.*, 1992). The success of strategies such as these depends on an understanding of how $pH_i$ is regulated within tumours *in vivo*, and what factors modulate this regulation. We have shown previously that under the acidic conditons that are likely to exist within solid tumours, the $Na^+/H^+$ exchanger is the major mechanism that is responsible for the regulation of $pH_i$ (Boyer & Tannock, 1992). Our finding that cells *in vivo* do not up-regulate their $Na^+/H^+$ exchanger implies that these cells are unlikely to be more resistant to the effects of drugs targeted against this antiporter than cells in a less acidic environment. We conclude therefore, that the $Na^+/H^+$ exchanger remains an appropriate target for anticancer therapy.

This study was supported by a grant (CA51033) from the National Institutes of Health, and by the Medical Research Council of Canada. Dr Boyer was supported by The Medical Research Council of Canada.

### References

BORON, W.F. (1989). Cellular buffering and intracellular pH. In *The Regulation of Acid-Base Balance*, Seldin, D.W. & Giebisch, G. (eds) pp. 33–56. Raven Press: New York.

BOYER, M.J. & TANNOCK, I.F. (1992). Regulation of intracellular pH in tumor cell lines: influence of microenvironmental conditions. *Cancer Res.*, **52**, 4441–4447.

CARLSSON, J. & ACKER, H. (1988). Relations between pH, oxygen partial pressure and growth in cultured spheroids. *Int. J. Cancer*, **42**, 715–720.

CASSEL, D., SCHARF, O., ROTMAN, M., CRAGOE, E.J Jr & KATZ, M.L. (1988). Characterization of $Na^+$-linked and $Na^+$-independent $Cl^-/HCO_3^-$ exchange systems in Chinese hamster lung fibroblasts. *J. Biol. Chem.*, **263**, 6122–6127.

CHAPLIN, D.J., DURAND, R.E. & OLIVE, P.L. (1985). Cell selection from a murine tumour using the fluorescent probe Hoechst 33342. *Br. J. Cancer*, **51**, 569–572.

CHAPLIN, D.J., TROTTER, M.J., DURAND, R.E., OLIVE, P.L. & MINCHINTON, A.I. (1989). Evidence for intermittent radiobiological hypoxia in experimental tumour systems. *Biomed. Biochim. Acta*, **48**, S255–S259.

CHU, G.L., WANG, Z., HYUN, W.C., PERSHADSINNGH, M.J., FULWYER, M.J. & DEWEY, W.C. (1990). The role of intracellular pH and its variance in low pH sensitization of killing by hyperthermia. *Radiat. Res.*, **122**, 288–293.

CRAGOE, E.J. Jr, WOLTERSDORF, O.W. Jr, BICKING, J.B., KWONG, S.F. & JONES, J.H. (1967). Pyrazine diuretics. II. N-amidino-3-amino-5-substituted 6-halopyrazinecarboxamides. *J. Med. Chem.*, **10**, 66–75.

DURAND, R.E. (1982). Use of Hoechst 33342 for cell selection from multicell systems. *J. Histochem. Cytochem.*, **30**, 117–122.

ERLICHMAN, C. & TANNOCK, I.F. (1986). Growth and characterization of multicellular tumor spheroids of human bladder carcinoma origin. *In vitro. Cell. Devel. Biol.*, **22**, 449–456.

FLIEGEL, L., SARDET, C., POUYSSEGUR, J. & BARR, A. (1991). Identification of the protein and cDNA of the cardiac $Na^+/H^+$ exchanger. *Febs. Letters*, **279**, 25–29.

GRASSL, S.M. (1991). Effect of chronic acid loading on rat renal basolateral membrane bicarbonate transport. *Biochim. Biophys. Acta*, **1061**, 226–234.

GRINSTEIN, S., COHEN, S. & ROTHSTEIN, A. (1984). Cytoplasmic pH regulation in thymic lymphocytes by an amiloride-sensitive $Na^+/H^+$ antiport. *J. Gen. Physiol.*, **83**, 341–369.

GRINSTEIN, S. & ROTHSTEIN, A. (1986). Mechanisms of regulation of the $Na^+/H^+$ exchanger. *J. Membrane Biol.*, **90**, 1–12.

HEDLEY, D.W. & JORGENSEN, H.B. (1989). Flow cytometric measurement of intracellular pH in B16 tumors: intercell variance and effects of pretreatment with glucose. *Exp. Cell. Res.*, **180**, 106–116.

HILDEBRANDT, F., PIZZONIA, J.H., REILLY, R.F., REBOUCAS, N.A., SARDET, C., POUYSSEGUR, J., SLAYMAN, C.W., ARONSON, P.S. & IGARASHI, P. (1991). Cloning, sequence, and tissue distribution of a rabbit renal $Na^+/H^+$ exchanger transcript. *Biochim. Biophys. Acta*, **1129**, 105–108.

HORIE, S., MOE, O., TEJEDOR, A. & ALPERN, R.J. (1990). Preincubation in acid medium increases Na/H antiporter activity in cultured renal proximal tubule cells. *Proc. Natl. Acad. Sci. USA*, **87**, 4742–4745.

HORIE, S., MOE, O., YAMAJI, Y., CANO, A., MILLER, R.T. & ALPERN, R.J. (1992). Role of protein kinase C and transcription factor AP-1 in the acid-induced increase in Na/H antiporter activity. *Proc. Natl. Acad. Sci. USA*, **89**, 5236–5240.

L'ALLEMAIN, G., FRANCHI, A., CRAGOE, E.J. Jr & POUYSSEGUR, J. (1984). Blockade of the $Na^+/H^+$ antiport abolishes growth factor-induced DNA synthesis in fibroblasts: structure-activity relationships in the amiloride series. *J. Biol. Chem.*, **259**, 4313–4319.

LYONS, J.C., KIM, G.E. & SONG, C.W. (1992). Modification of intracellular pH and thermosensitivity. *Radiat. Rev.*, **129**, 79–87.

MAIDORN, R., CRAGOE, E.J. Jr & TANNOCK, I.F. (1993). Therapeutic potential of analogues of amiloride: inhibition of the regulation of intracellular pH as a possible mechanism of tumour selective therapy. *Br. J. Cancer*, **67**, 297–303.

MINCHINTON, A.I., DURAND, R.E. & CHAPLIN, D.J. (1990). Intermittent blood flow in the KHT sarcoma-flow cytometry studies using Hoechst 33342. *Br. J. Cancer*, **62**, 195–200.

MOE, O.W., MILLER, R.T., HORIE, S., CANO, A., PREISIG, P.A. & ALPERN, R.J. (1991). Differential regulation of Na/H antiporter by acid in renal epithelial cells and fibroblasts. *J. Clin. Invest.*, **8**, 1703–1708.

NEWELL, K., WOOD, P., STRATFORD, I. & TANNOCK, I. (1992). Effects of agents which inhibit the regulation of intracellular pH on murine solid tumours. *Br. J. Cancer*, **66**, 311–317.

REILLY, R.F., HILDEBRANDT, F., BIEMESDERFER, D., SARDET, C., POUYSSEGUR, J., ARONSON, P.S., SLAYMAN, C.W. & IGARASHI, P. (1991). cDNA cloning and immunolocalization of a $Na^+$-$H^+$ exchanger in LLC-PK1 renal epithelial cells. *Am. J. Physiol.*, **261**, F1088–F1094.

REINERTSEN, K.V., TONNESSEN, T.I., JACOBSEN, J., SANDVIG, K. & OLSNES, S. (1988). Role of chloride/bicarbonate antiport in the control of cytosolic pH: cell-line differences in activity and regulation of antiport. *J. Biol. Chem.*, **263**, 11117–11125.

RINK, T.J., TSIEN, R.Y. & POZZAN, T. (1982). Cytoplasmic pH and free $Mg^{2+}$ in lymphocytes. *J. Cell Biol.*, **95**, 189–196.

ROOS, A. & BORON, W.F. (1981). Intracellular pH. *Physiol. Rev.*, **61**, 296–434.

ROTIN, D., ROBINSON, B. & TANNOCK, I.F. (1986). Influence of hypoxia and an acidic environment on the metabolism and viability of cultured cells: potential implications for cell death in tumors. *Cancer Res.*, **46**, 2821–2826.

SARDET, C., FRANCHI, A. & POUYSSEGUR, J. (1989). Molecular cloning, primary structure, and expression of the human growth factor-activatable $Na^+/H^+$ antiporter. *Cell*, **56**, 271–280.

SCHWARTZ, M.A., CRAGOE E.J. Jr & LECHENE, C.P. (1990). pH regulation in spread cells and round cells. *J. Biol. Chem.*, **265**, 1327–1332.

SUTHERLAND, R.M. (1988). Cell and environment interactions in tumor microregions: the multicell spheroid. *Science*, **240**, 177–184.

TANNOCK, I.F. & ROTIN, D. (1989). Acid pH in tumors and its potential for therapeutic exploitation. *Cancer Res.*, **49**, 4373–4384.

THOMAS, J.A., BUCHSBAUM, R.N., ZIMNIAK, A. & RACKER, E. (1979). Intracellular pH measurements in Ehrlich ascites tumor cells utilizing spectroscopic probes generated *in situ*. *Biochemistry*, **18**, 2210–2218.

TONNESSEN, T.I., SANDVIG, K. & OLSNES, S. (1990). Role of $Na^+$-$H^+$ and $Cl^-/HCO_3^-$ antiports in the regulation of cytosolic pH near neutrality. *Am. J. Physiol.*, **258**, C1117–C1126.

TSE, C.M., MA, A.I., YANG, V.W., WATSON, A.J., LEVINE, S., MONTROSE, M.H., POTTER, J., SARDET, C., POUYSSEGUR, J. & DONOWITZ, M. (1991). Molecular cloning and expression of a cDNA encoding the rabbit ileal villus cell basolateral membane $Na^+/H^+$ exchanger. *EMBO J.*, **10**, 1957–1967.

VAUPEL, P., KALLINOWSKI, F. & OKUNIEFF, P. (1989). Blood flow, oxygen and nutrient supply, and metabolic microenvironment of human tumors: a review. *Cancer Res.*, **49**, 6449–6465.